# Construction and characterisation of a recombinant fowlpox virus that expresses the human papilloma virus L1 protein

**DOI:** 10.1186/1479-5876-9-190

**Published:** 2011-11-04

**Authors:** Carlo Zanotto, Eleana Pozzi, Sole Pacchioni, Massimiliano Bissa, Carlo De Giuli Morghen, Antonia Radaelli

**Affiliations:** 1 Department of Medical Pharmacology, University of Milan, 20129 Milan, Italy; 2 Department of Pharmacological Sciences, University of Milan, 20133 Milan, Italy; 3 Cellular and Molecular Pharmacology Section, Institute of Neurosciences, University of Milan, 20129 Milan, Italy

## Abstract

**Background:**

Human papilloma virus (HPV)-16 is the most prevalent high-risk mucosal genotype. Virus-like-particle (VLP)-based immunogens developed recently have proven to be successful as prophylactic HPV vaccines, but are still too expensive for developing countries. Although vaccinia viruses expressing the HPV-16 L1 protein (HPV-L1) have been studied, fowlpox-based recombinants represent efficient and safer vectors for immunocompromised hosts due to their ability to elicit a complete immune response and their natural host-range restriction to avian species.

**Methods:**

A new fowlpox virus recombinant encoding HPV-L1 (FP_L1_) was engineered and evaluated for the correct expression of HPV-L1 *in vitro*, using RT-PCR, immunoprecipitation, Western blotting, electron microscopy, immunofluorescence, and real-time PCR assays.

**Results:**

The FP_L1 _recombinant correctly expresses HPV-L1 in mammalian cells, which are non-permissive for the replication of this vector.

**Conclusion:**

This FP_L1 _recombinant represents an appropriate immunogen for expression of HPV-L1 in human cells. The final aim is to develop a safe, immunogenic, and less expensive prophylactic vaccine against HPV.

## Background

Cervical cancer caused by human papilloma virus (HPV) is the second leading cause of malignancies in women worldwide, and the first in developing countries. Persistent infection with one of the approximately fifteen high-risk oncogenic HPV types can cause the onset of cervical intraepithelial neoplasia and progression to invasive cervical cancer [1]. Approximately 500,000 cases are diagnosed every year, resulting in 274,000 deaths [2,3].

The absence of specific antiviral drugs has stimulated the search for prophylactic vaccines against HPV-16 and HPV-18, which are the HPV genotypes that are most commonly associated with the disease. In particular, HPV-16 is by far the most prevalent high-risk mucosal genotype, and, as it is present in around 50% of all cervical cancers [1,4,5], it has been the focus of many recent vaccine developments.

Although therapeutic vaccines are of high priority for the control of progression to neoplasia in subjects who are already infected, prophylactic vaccines are important to limit the diffusion of infection. They are therefore the best choice for intervention against HPVs, as they can neutralise the incoming virus and prevent disease progression.

The preparation of a new vaccine has been hampered for a long time because of the restricted host range of the virus and its selective tropism for differentiated squamous epithelium, which makes its growth in cell culture difficult. Expression of the L1 major capsid protein of HPV-16 (HPV-L1) in eucaryotic cells results in the formation of empty virus capsids (virus-like particles; VLPs) [6] which are very similar to native virions, show icosahedral symmetry, and can enter cells like infectious virions. In particular, VLPs have proven to be successful as prophylactic bivalent [7] and quadrivalent [8] HPV vaccines in women, by eliciting virus-neutralising antibodies in large randomized controlled clinical trials [9-11]. They have also shown higher immunogenicity than capsomers, although similar titres of neutralising antibodies can be induced using strong immune adjuvants [12]. VLPs enter the MHC-II processing pathway and they can induce a CD4-restricted T-cell response, which is important for both robust antibody and memory responses, as has been summarized in different reviews [13-15]. Different studies also suggest that VLPs can also deliver foreign protein to the MHC-I pathway to elicit cytotoxic T-cell responses [16-18]. However, VLPs are still too expensive for less-developed countries, they are not thermostable, and they need to be delivered by injection.

Immunisation with vaccinia virus recombinants expressing HPV genes or other viral antigens has been demonstrated to be a good candidate for stimulation of the immune system [19]. However, their use has raised safety concerns due to severe collateral effects in immunocompromised individuals [20]. VLPs have also been produced in plant in various studies [21,22], and also as a chimeric L1 fused with cytotoxic E6 epitopes [23].

The avipox viruses, and the fowlpox virus (FP) in particular, represent alternative vectors due to their natural host-range restriction to avian species [24,25], to their correct expression of transgenes in mammalian cells, and to their elicitation of a long-lasting immune response in the vaccinated hosts [26,27]. Compared to VLP-based HPV vaccines that are produced by yeast or baculovirus, and that mainly induce humoral responses, FP-based recombinants elicit a complete and more effective immunity. Moreover, as these FP-based recombinants do not immunologically cross-react with vaccinia viruses, they can be administered to previously vaccinia-virus-experienced individuals, thus circumventing neutralisation by vector-generated immunity [28]. These viruses have thus acquired an important role in the development of novel vaccines against human diseases, as they may represent safer vectors than wild-type or attenuated vaccinia viruses [24,29].

In the present study, a new FP recombinant encoding HPV-L1 (FP_L1_) was engineered and evaluated for its correct expression *in vitro*, with the final aim of developing a prophylactic vaccine. This recombinant should express foreign genes intracellularly and allow cross-presentation of the antigen in association with both MHC class I and II complexes, thus eliciting a complete humoral and cellular protective immunity.

## Methods

### Cells

CaSki cells carrying multiple copies of integrated HPV-16 DNA, green monkey kidney (Vero) cells, and MRC-5 human lung fibroblasts were grown in Dulbecco's modified Eagle's medium (DMEM) supplemented with 10% heat-inactivated calf serum (Gibco/Invitrogen, Carlsbad, CA, USA), 100 U/ml penicillin and 100 μg/ml streptomycin (P/S). Specific-pathogen-free primary chick embryo fibroblasts (CEFs) were grown in DMEM with 5% heat-inactivated calf serum, 5% tryptose phosphate broth (TPB; Difco Laboratories, Detroit, MI, USA), and P/S.

### Production of HPV-16 L1

The pQE30 expression plasmid (Qiagen, Valencia, CA, USA) engineered to contain the L1 gene of HPV-16 [30] (pQE30-L1-NLS-His) was kindly supplied by C. Giorgi (Istituto Superiore di Sanità, Rome, Italy). After its cloning into JM109 bacterial cells, this engineered plasmid was used for the production of the RGS/L1/His-tagged protein, according to the manufacturer instructions (Qiagen), with minor modifications. Briefly, JM109/pQE30-L1-NLS-His bacterial cells were lysed in phosphate lysis buffer (300 mM NaCl, 1% Triton X-100, pH 8, prepared in buffer A: 10 mM Tris, 100 mM Na_2_HPO_4_, 6 M guanidine-HCl, pH 8). After clarification for 30 min at 17,000 × *g *at 4°C, the supernatant containing the HPV-L1 preparation was supplemented with 1% Triton X-100/20 mM imidazole, pH 8, in buffer A. This was then incubated with Ni-NTA agarose resin (Qiagen) for 30 min at room temperature. After washing once with 1% Triton X-100 in buffer A, twice with buffer A, and multiple times with buffer C (100 mM Na_2_HPO_4_, 10 mM Tris, 8 M Urea, pH 6.3) up to a final optical density of 0.013, the protein was eluted into different fractions with 1 M imidazole, pH 8. After their analysis by 15% PAGE, the fractions enriched in the recombinant HPV-L1 were pooled. The HPV-L1 was dialysed overnight at 4°C using a slide-A-lyser cassette (10 kDa MW cut-off, Pierce, Rockford, IL, USA), and soaked in dialysis buffer (25 mM Tris-HCl, 100 mM NaCl). The protein was quantified, stored at -80°C and used as a control in the Western blotting.

### Construction of the recombination plasmid

The L1 gene of HPV-16 was amplified from the pUF3/L1 plasmid that contained the humanised HPV-L1 gene sequence (1,518 bp; accession number: AJ313179) [31] kindly supplied by M. Mueller (German Cancer Research Center, Heidelberg, Germany). This was inserted into the multicloning site (MCS) downstream of the early/late VVH6 promoter [32,33] of the pFP-MCS-GFP plasmid. The enhanced GFP (green fluorescent protein) gene derived from pEGFP-N1 (BD Biosciences Clontech Laboratories, Inc., Mountain View, CA, USA) is therefore located downstream of the L1 gene, but in the reverse orientation and under the control of the synthetic SP promoter [34] (a gift from A. Siccardi, HSR, Milan, Italy). The L1 and GFP genes are both inside the two arms of the 3-β-hydroxysteroid dehydrogenase 5-delta 4 isomerase gene (DH) fowlpox gene, used for site-specific *in-vitro *recombination [35]. The DNA sequence encoding the complete L1 region of HPV-16 was amplified using the V178 (5'-GCC-GCG-CCC-GGG-AAG-CTT-ATG-AGC-CTG-TGG-CTG-CCC-AGC-GAG-3') and V179 (5'-GCC-GCG-GTC-GAC-AAG-CTT-TCA-CAG-CTT-CCT-CTT-CTT-CC-3') primers. The amplification was carried out starting from 250 ng DNA in a final volume of 50 μl, in a mixture containing 1 μM of each primer, 200 μM of each dNTP, 3 μM MgCl_2_, 2.0 mM MgSO_4_, and 0.025 U/μl Pwo DNA polymerase (Boehringer Mannheim, Indianapolis, IN, USA). The PCR conditions were: 95°C for 45 s, followed by 30 cycles at 95°C for 30 s, 70°C for 30 s, 72°C for 80 s, and 72°C for 7 min (PTC-200 thermocycler; MJ Research, Waltham, MA, USA). The HPV-L1 gene was first cloned into the pCR-BluntII-TOPO plasmid (Invitrogen, Carlsbad, CA, USA), which allows the insertion of blunt-ended sequences. The pCR-BluntII-TOPO/L1 was then cut out with HindIII (Fermentas, M-Medical, Milan, Italy) and ligated into the pFP recombinant vector, which had been previously linearised with HindIII for 1 h at 37°C. Ligation was carried out overnight at 16°C in 10 μl, using 1 U T4 DNA ligase (USB, Amersham-Pharmacia Biotech AB, Uppsala, Sweden) and 100 ng of the insert at an insert:vector molar ratio of 3:1. After transformation of supercompetent ECL bacterial cells, the colonies were screened by amplifying the HPV-L1 gene, and further analysed by digestion with the NruI/KpnI and HindIII/EcoRI restriction enzymes, to verify the correct orientation of the insert. The cloned recombinant plasmid was purified (Qiagen, Hilden, Germany) and sequenced (Genenco, MMedical, Milan, Italy) to exclude any possible mutation arising from PCR amplification. This cloned recombinant plasmid is henceforth referred to as pFP_L1 _(9,726 bp).

### In-vitro recombination to generate the FP_L1 _recombinant

The FP_L1 _recombinant was obtained by *in-vitro *recombination in specific-pathogen-free primary CEFs, as described previously [35,36], using the wild-type FP virus (FPwt) and pFP_L1_. Briefly, the CEFs were infected with 0.5 PFU/cell FPwt in DMEM containing 2% foetal calf serum, and, after a 4-h incubation at 37°C, they were transfected by calcium phosphate precipitation using 125 μg pFP_L1 _DNA in 1 ml of a mixture containing 125 μM CaCl_2 _in 40 mM HEBS, pH 7, 300 mM NaCl, 1.4 mM Na_2_HPO_4_, 10 mM KCl, and 12 mM dextrose. Two days after the infection, the FP virus was released from the cells by three freeze-thaw cycles and used for further infection and selection of the recombinants. Recombinant plaques were identified by autoradiography after hybridisation with a [^32^P]-labelled HPV-L1 probe, and then subjected to multiple cycles of plaque purification. One clone was selected for correct and highest expression of the HPV-L1 gene by Western blotting. The FP_L1 _recombinant was amplified in CEFs and purified on a sucrose gradient, as described previously [33].

### mRNA transcript expression in replication-restrictive Vero cells

The expression of the HPV-L1 gene was first determined by RT-PCR, after infecting Vero cells with 5 PFU of FP_L1 _recombinant. The experiments were performed in duplicate. RNA was extracted 1 day post-infection (p.i.) and every 3 days for 21 days, using Trizol LS (Gibco) to determine the level of HPV-L1 transcripts in non-permissive mammalian cells. The mRNAs from all of the samples were treated with 10 U RNase-free DNase I (Roche Diagnostics, Indianapolis, IN, USA) for 4 h at 37°C, to eliminate any cellular or viral DNA, then precipitated with 100% ethanol in the presence of 100 mM Na acetate. After washing in 75% ethanol and resuspension in diethylpyrocarbonate-treated water, RT-PCR was carried out using the Access RT-PCR System kit (Promega, Madison, WI, USA). Briefly, 50 ng RNA from each sample was used in a final volume of 20 μl, in the presence of 1 mM of each primer, 200 mM of each dNTP, 0.1 U/ml Tfl DNA polymerase, 0.1 U/ml AMV reverse transcriptase (AMV-RT), and 2.0 mM MgSO_4_. Control samples without AMV-RT were also prepared. The presence of the HPV-L1 transcript was verified using the V191 (5'-CGA-CAC-CAG-CTT-CTA-CAA-C-3') and V190 (5'-TGT-TGA-ACA-GGT-GCC-TC-3') primers. Human β-actin was also amplified as an internal control, using the V84 (5'-CTG-ACT-ACC-TCA-TGA-AGA-TCC-T-3') and V85 (5'-GCT-GAT-CCA-CAT-CTG-CTG-GAA-3') primers, and a band of 518 bp was obtained. Mock-infected Vero cells were used as a negative control. The reverse transcriptase reaction was carried out at 48°C for 45 min, followed by 2 min at 94°C. PCR amplification was performed for 35 cycles at 94°C for 30 s, 60°C for 30 s, and 68°C for 1 min, followed by a final incubation at 68°C for 7 min. For human β-actin, the RNA (50 ng) amplification was carried out as described above, but with 1 mM MgSO_4_. The thermal profile was at 48°C for 45 min followed by 94°C for 2 min and 40 cycles at 94°C for 30 s, 58°C for 30 s, 68°C for 1 min, and with a final extension of 68°C for 7 min. The PCR products were run on 1% agarose gels and the gel images were acquired using a Speedlight Platinum apparatus (Lightools Research, Encinitas, CA, USA). The RT-PCR products were quantified using the ImageJ software [37].

### Radioimmunoprecipitation analysis

The cells were infected with 5 PFU/ml in DME methionine-, cystine-, and L-glutamine-free medium (DMEM met^-^, cys^-^, glut^-^, MP Biomedicals Inc, DBA, Milan, Italy). After 2 h, 20 μCi/ml [^35^S]-methionine and [^35^S]-cysteine were added, using the same medium supplemented with 2% dialysed foetal bovine serum (Gibco). Sixteen hours p.i., the cells in 2 ml of medium were harvested by resuspending them in 1 ml lysis buffer (150 mM NaCl, 1 mM EDTA, 10 mM Tris-HCl, pH 7.4, 0.2 mg/ml PMSF, 1% NP40, 0.01% sodium azide) and 0.6 TIU aprotinin (Sigma, St Louis, MO, USA) per Petri dish, with scraping into microcentrifuge tubes (Eppendorf, Milan, Italy). The lysate was clarified by centrifugation at 9,000 × *g *for 20 min at 4°C. Immunoprecipitation was performed either with 2 μl anti-HPV-L1 preadsorbed polyclonal mouse serum (C. Giorgi) or with a monoclonal antibody (CamVir-1; BD Biosciences, San Diego, CA, USA). Proteins were resolved using 12.5% SDS-PAGE, and fluorographed.

### Western blotting

To determine whether HPV-L1 was expressed correctly by the FP_L1 _recombinant, Vero cells were infected with 5 PFU/cell FP_L1 _recombinant and examined by Western blotting. The pelleted cells were lysed in sample buffer (50 mM Tris, pH 6.8, 10% SDS, 1.5% dithiothreitol, 0.05% bromophenol blue), boiled for 5 min, and loaded onto polyacrylamide gels. After running through a 4% stacking gel for 1 h at 20 mA and through a 15% running gel for 1.5 h at 40 mA, the proteins were transferred onto 0.2 μm nitrocellulose membranes (Bio-Rad Laboratories, Milan, Italy) for 1 h at 100 V in cold transfer buffer (150 mM glycine, 20 mM Tris, 20% methanol) using a transblot apparatus (Bio-Rad). Soon after the protein transfer to the nitrocellulose, the positions of protein markers (Precision Plus Protein™ All blue standards, Bio-Rad) (5 μl/gel) were marked on the nitrocellulose. The nitrocellulose membranes were incubated for 30 min in 0.5% glutaraldehyde (Polysciences, Inc., Warrington, PA, USA), washed in H_2_O, and incubated for 1 h with 5% skimmed milk (Merck, West Point, PA, USA) in Ca^2+^-free and Mg^2+^-free phosphate-buffered saline (PBS^-^). After three rinses in wash buffer (20% Tween in PBS^-^), the nitrocellulose membrane was incubated overnight at 4°C with a 1:5,000 dilution of the specific anti-HPV-L1 CamVir-1 antibody (BD Biosciences). The washing was followed by a 1-h incubation with a goat anti-mouse horseradish peroxidase antibody (1:10,000 dilution; Dako Cytomation), and 2-h washes, before the HPV-L1 was revealed using the ECL system (GE Healthcare, Buckinghamshire, UK). The densitometric analysis of the Western blotting bands of the L1 protein was performed using the ImageJ software [37].

### Immunofluorescence

The expression of HPV-L1 was also examined by immunofluorescence. Vero cells were seeded at a density of 5 ×10^5^/35-mm^2 ^dish on sterile glass coverslips, before infection with 5 PFU/cell FP_L1 _recombinant at 37°C for 1 h. After a 6-h incubation at 37°C in DMEM supplemented with 2% foetal calf serum and P/S, the cells were washed twice with PBS^-^, fixed with 2% paraformaldehyde (Polysciences) in PBS^- ^for 10 min at room temperature, followed by 100% cold acetone for 5 min at 4°C. The samples were incubated with either monoclonal CamVir-1 (BD Biosciences, 1:100) or mouse (C. Giorgi, 1:50) or rabbit (R20, from our laboratory, 1:500) polyclonal anti-HPV-L1 antibodies, followed by a 1:100 FITC antiserum (Cappel, MP Biomedicals, Inc., Aurora, OH, USA). The R20 antibodies were obtained by multiple inoculations of one rabbit with the FP_L1 _recombinant. Before use, serum from R20 was immmunoadsorbed overnight with FPwt-infected Vero cells to remove antibodies raised against the vector. FPwt-infected Vero cells were used as a negative control, while CaSki and HeLa cells were used as positive controls. Samples were viewed under a Zeiss Axioskop fluorescence microscope.

### Transmission electron microscopy

Confluent Vero, MRC-5, and CEFs cells were infected with the FP_L1 _recombinant and FPwt at 5 PFU/cell for 1 h at 37°C. On days 1 and 3 p.i., the cells were harvested for transmission electron microscopy (TEM). After centrifugation at 1,000 × *g *for 10 min at room temperature, the cells were fixed in 2.5% glutaraldehyde (Polysciences) in 0.1 M Na cacodylate buffer, pH 7.4, for 1 h at 4°C. They were then rinsed twice, and post-fixed in cacodylate-buffered 1% OsO_4 _at 4°C for 1 h. The specimens were dehydrated through a series of graded ethanol solutions and propylene oxide, and embedded in Poly/Bed 812 resin mixture. Ultrathin sections were obtained using a Sorvall MT2B ultramicrotome equipped with a diamond knife, and they were stained with water-saturated uranyl acetate and 0.4% lead citrate in 0.1 M NaOH. VLPs from the commercial HPV Gardasil^® ^vaccine were negative-stained with water-saturated uranyl-acetate and used as a control. The specimens were viewed with a Philips CM10 electron microscope.

### Real-time PCR

RNA was extracted from Vero cells infected with 2 PFU/cell FP_L1 _recombinant, and harvested on days 1, 2, 7, 10, 14, 18 and 22 p.i.. The cells were rinsed twice with PBS^-^, scraped from the Petri dishes with a rubber policeman, and centrifuged at 1,500 × *g *for 5 min. After lysis, the RNAs were extracted using the RNeasy mini kit (Invitrogen, Carlsbad, CA, USA), according to the manufacturer instructions. Reverse-transcriptase reactions were performed in a final volume of 50 μl, using 3 μg RNA and the High Capacity cDNA Archive kit (PE Applied Biosystems, Foster City, CA, USA). The reactions were performed at 25°C for 10 min, followed by 48°C for 30 min, and 95°C for 5 min. The cDNA (5 μl) was then added to each well of a MicroAmp Optical 96-well reaction plate (Applera, PE Applied Biosystems) in the presence of 2× Power SYBRA green master mix, and forward V392 (5'-GGA-GTA-CGA-CCT-GCA-GTT-CAT-CT-3') and reverse V393 (5'-CTG-CGT-TCA-TCG-TGT-GGA-TGT-3') primers to identify the HPV-L1 gene, in a final volume of 25 μl. The primers V385 (5'-AGC-AAA-GAC-CCC-AAC-GAG-AA-3') and V386 (5'-GGC-GGC-GGT-CAC-GAA-3') were used for the *GFP *gene, V380 (5'-TGG-AGA-TGA-ACT-CGG-ACC-TC-3') and V381 (5'-CGA-CCA-CCA-CCA-ACT-TCA-A-3') for the housekeeping *RPS7 *human gene. FPwt-infected Vero cells and Vero cells transfected for 24 h with the pUF3/HPV-L1 plasmid (kindly supplied by M. Mueller, German Cancer Research Centre, Heidelberg, Germany) or the pEGFP plasmid were used as negative and positive controls, respectively. All of the reactions were performed in an ABI PRISM 7700 apparatus (PE Applied Biosystems). The PCR conditions were 50°C for 2 min, 95°C for 15 min, followed by 40 cycles at 95°C for 15 s and 60°C for 1 min. A 20-min dissociation protocol was also applied. Two different analyses were performed and the copy numbers of the HPV-L1 and GFP transcripts were calculated using the comparative C_t _method (also known as the 2^-[delta] [delta]Ct ^method), where [delta] C_t, sample _= C_t,L1 _- C_t, RPS7_, [delta] C_t, sample _is the C_t _value for any sample normalised to the RPS7 endogenous housekeeping transcripts and [delta] [delta]C_t _= [delta]C_t, sample _- [delta]C_t, wt_. In all of the samples, the 2^-[delta] [delta]Ct ^refers to an N-fold increase of HPV-L1/GFP copy number relative to the control.

## Results

### FP_L1 _recombinant expresses the mRNA up to day 21 p.i. in non-permissive mammalian cells

**Figure 1:**
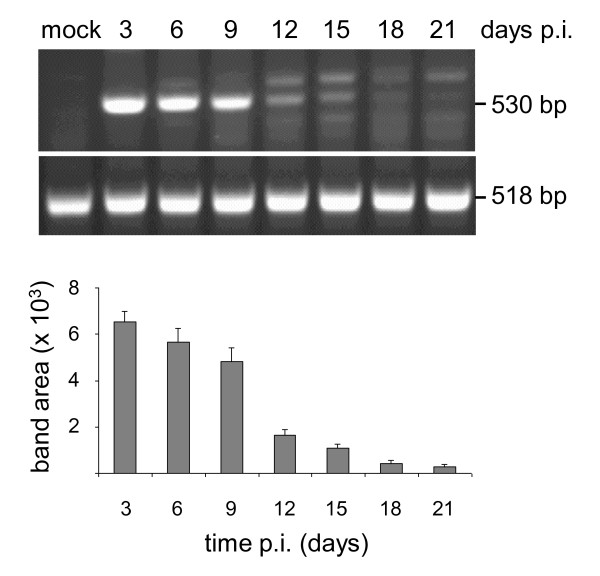
**Expression of the HPV-L1 transcript over time by RT-PCR**. After infecting Vero cells with the FP_L1_ recombinant, in duplicate, the expression of the transgene was evaluated every 3 days. The expression levels, as determined by densitometric analysis, extended up to day 21 p.i. (530 bp). The data are expressed as means (±standard deviation). Amplification of β-actin RNA (518 bp) is also shown. Mock-infected cells were always negative, as were cells infected with FPwt or amplified in the absence of AMV-RT (data not shown).

The expression of the HPV-L1 transgene after infection by the FP_L1 _recombinant was tested over time by RT-PCR. The mRNA isolated from infected Vero cells showed that the gene carried by the FP_L1 _recombinant amplified as a band of 530 bp (Figure 1). In particular, HPV-L1 transgene expression was detectable from 3 days p.i., and extended up to day 21 p.i.. The expression levels were determined by densitometric analysis, and they showed that a very high level of expression was maintained up to day 9 p.i., which then constantly decreased. The similar levels of amplification of β-actin RNA (518 bp) over time confirmed the reliability of these differences in HPV-L1 expression. Mock-infected cells were always negative, as were cells infected with FPwt and those amplified in the absence of AMV-RT (data not shown).

### HPV-L1 is expressed in mammalian cells, although at low levels

**Figure 2:**
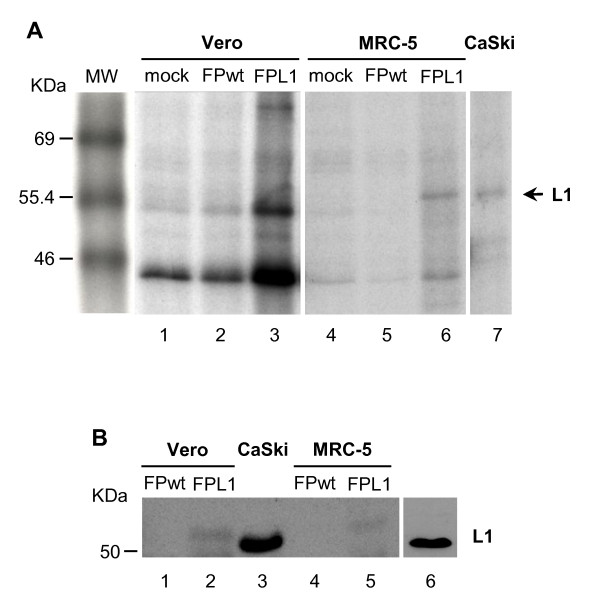
**Transcript expression by immunoprecipitation (A) and Western blotting (B)**. HPV-L1 expression was tested in cells infected with 5 PFU/cell of the FP_L1 _recombinant virus. By immunoprecipitation, HPV-L1 was detected only in MRC-5 cells (A, lane 6), whereas by Western blotting HPV-L1 expression was seen not only in MRC-5 cells (B, lane 5), but also in Vero cells (B, lane 2). Both immunoprecipitation and Western blotting were performed using the CamVir-1 monoclonal antibody. HPV-L1 produced by an engineered bacterial vector was used as a molecular weight marker (lane 6) and CaSki cells as a positive control (lane 3). No specific proteins were seen in mock-infected (A, lanes 1, 4) and FPwt-infected (A, lanes 2, 5; B, lanes 1, 4) cells or in replication-permissive CEFs (data not shown). HPV-L1 (B, lane 6) and CaSki cells (A, lane 7; B, lane 3), used as positive controls, also showed a clear 55-kDa band.

To determine whether HPV-L1 transcripts were followed by the synthesis of the corresponding viral proteins, immunoprecipitation was performed on Vero, MRC-5, and CEFs cells (Figure 2A). HPV-L1 was detected only in MRC-5 cells (Figure 2A, lane 6), using either monoclonal or rabbit polyclonal antibodies. In the FP_L1_-infected Vero cells, protein expression was not detectable (Figure 2A, lane 3) as well as in CEFs (data not shown), and in mock-infected (Figure 2A, lanes 1 and 4) and FPwt-infected (Figure 2A, lanes 2 and 5) cells. These data prompted us to confirm these results by Western blotting with the same cell lines (Figure 2B). The results showed expression of HPV-L1 not only in MRC-5 cells (Figure 2B, lane 5) but also in Vero cells infected by the FP_L1 _recombinant (Figure 2B, lanes 2, 55-58 kDa). The densitometric analysis of the Western blotting bands of the L1 protein produced by the FP_L1 _recombinant was compared to the band of a known amount of purified L1 protein used as a standard; this showed that 10^7 ^Vero and MRC-5 cells produced 42 ng and 52 ng of protein, respectively. HPV-L1 produced by an engineered bacterial vector was used as a molecular-weight marker (Figure 2B, lane 6) and CaSki cells as a positive control (Figure 2B, lane 3). No specific bands were seen when Vero cells were infected with FPwt (Figure 2B, lanes 1 and 4) or in replication-permissive CEFs (data not shown). It is worth noting that although different monoclonal and polyclonal antibodies were used to determine the expression of HPV-L1, only the monoclonal BD antibody showed specificity and high avidity in all of the different tests.

### Immunofluorescence results

**Figure 3:**
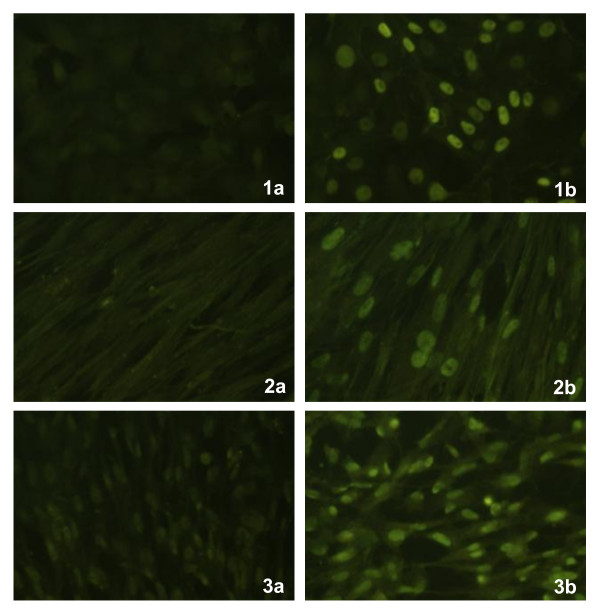
**Heterologous protein expression by immunofluorescence**. In all the different, either non-permissive (Vero and MRC-5 cells, 1b, 2b) or permissive (CEFs, 3b) cell types, immunofluorescence was detected at the nuclear level, using the CamVir-1 antibody. No fluorescence was seen in the respective different cell types infected with FPwt (1a, 2a, 3a). As expected, the CaSki and HeLa cells used as positive controls showed clear nuclear immunofluorescence (data not shown).

To determine the localisation of HPV-L1, immunofluorescence (Figure 3) was performed on Vero, MRC-5, and CEFs cells infected with the FP_L1 _recombinant. In all these different cell types, which were either non-permissive (Vero and MRC-5, Figure 3, 1b, 2b) or permissive (CEFs, Figure 3b) for replication, immunofluorescence was detected at the nuclear level using monoclonal or polyclonal antibodies. No fluorescence was seen in the respective different cell types infected with FPwt (Figure 3, 1a, 2a, 3a). As expected, CaSki and HeLa cells used as positive controls showed clear nuclear immunofluorescence (data not shown).

### Only limited amounts of VLPs are found in Vero and MRC-5 cells

**Figure 4:**
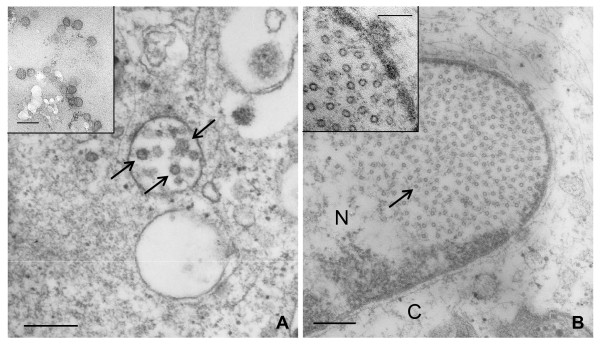
**VLP production in Vero and MRC-5 cells**. Cells infected with the FP_L1 _recombinant were fixed 3 days p.i., and VLP formation was examined at ultrastructural level by electron microscopy. Complete VLPs (T7, 72 pentamers) were found in Vero cells mainly enclosed in pseudo-vacuoles (A). Unassembled T1-particles (12 pentamers) were present in the nuclei of MRC-5 cells (B). No VLPs were detected in FP_L1_-infected CEFs (data not shown). Negative-stained VLPs from the commercial HPV Gardasil^® ^vaccine (inset of panel A) were used as a control. An enlargement of the nuclear T1 particles is shown in the inset of panel B. Bar in Panels A and B: 300 nm. Bar in the insets: 100 nm.

Structural capsid proteins can self-assemble into VLPs spontaneously. Non-permissive Vero (Figure 4A) and MRC-5 (Figure 4B) cells, as well as permissive CEFs (not shown), were infected with the FP_L1 _recombinant, and VLP formation was examined at the ultrastructural level by electron microscopy. In both Vero and MRC-5 cells, FP_L1 _recombinant virions were seen attached to the cell membrane (data not shown). Complete VLPs (T7, 72 pentamers) were seen in Vero cells at different times p.i., although in limited amounts and mainly enclosed in pseudo-vacuoles (Figure 4A). These pseudo-vacuoles seem to be the result of tangential sections of invaginations of the cell membrane, as shown by the unstructured background characteristics of the extracellular environment. In MRC-5 cells, only unassembled particles (T1, 12 pentamers) were seen in the nucleus (Figure 4B), whereas they could not be detected in FP_L1_-infected CEFs (data not shown).

### GFP is well expressed over time

**Figure 5:**
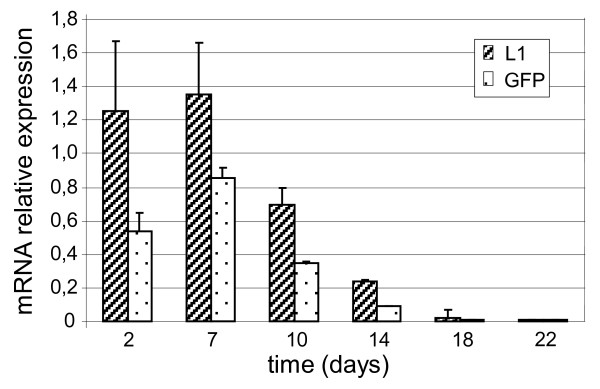
**Expression of HPV-L1 and GFP transcripts over time using real-time PCR in restrictive mammalian cells**. After infecting Vero cells with 2 PFU/cell of the FP_L1 _recombinant, quantitative expression of the HPV-L1 and GFP transgenes was evaluated every 3 days for 22 days. The maximum expression level for both HPV-L1 and GFP were reached on day 7 p.i., and GFP mRNAs were always highly expressed. The copy number of HPV-L1/GFP mRNAs, normalised to the RPS7 endogenous housekeeping transcripts, refers to the N-fold increase *vs *control mRNA at each time point. The data are expressed as means (±standard deviation) from two independent analyses.

To determine whether the limited amount of VLPs can be ascribed to the interaction of the HPV-L1 and GFP genes driven by the VVH6 and SP promoters, respectively, a quantitative comparison between the HPV-L1 and GFP transcripts was performed over 3 weeks by real-time PCR at different days p.i. (Figure 5). The GFP mRNAs were expressed over time at a good level as well as the HPV-L1 transcripts, reaching their maximum expression level on day 7 p.i..

## Discussion

HPV-16 infection is the major risk factor for the development of cervical neoplasia and invasive cancer [38], and its evidence as the primary aetiologic agent of cervical cancer has been shown across a wide variety of epidemiologic and experimental studies [39]. The expression of the major L1 capsid protein of papillomavirus (HPV-L1) leads to self-assembly of VLPs, that display conformational epitopes recognised by neutralizing monoclonal antibodies [40], as has been demonstrated using baculovirus-transduced insect cells [41,42] or yeast [43-45]. The preventive VLP-based vaccines, that have been recently developed (Gardasil^®^, Merck; Cervarix^®^, Glaxo SmithKline Biologicals) and approved for immunisation programmes in several countries [46], are effective in eliciting protective immunity through induction of a prominent humoral response [6,13,14,47]. However, their cost is unaffordable by the healthcare systems of less-developed countries, where the incidence of HPV-associated disease is extremely high, and the vaccine is most needed, also because HIV-positive subjects are unable to clear HPV infections. Also, as seen for non-replicating vaccines, VLPs are endocytosed by antigen-presenting cells, mainly presented to CD4^+ ^T-lymphocytes in association with MHC class II molecules, and require boost inoculations to maintain high antibody levels and immune cell memory. Recombinant live viral vaccines driving HPV transgene expression represent an attractive alternative as they might induce humoral and cellular immune responses and a long-lasting immunity without the need for numerous recalls.

In the present study, we have described the construction, transcription and protein expression of a new FP recombinant containing the humanised L1 gene of HPV-16, and we have demonstrated that: (1) HPV-L1 is correctly expressed in mammalian cells, as demonstrated by RT-PCR, immunoprecipitation, Western blotting, and real-time PCR; (2) in spite of the abortive replication of the FP virus vector in mammalian cells, the transcription of HPV-L1 is long lasting; (3) HPV-L1 is localised at the nuclear level, as seen by immunofluorescence; (4) L1 self-assembles both as T1 particles and as VLPs, and it can be found both in non-human and human primate cells.

The long-lasting expression of the HPV-L1 transcripts in mammalian cells confirms the possibility for a non-replicating recombinant vector to induce a good immune response [48]. As the HPV-L1 was found by all of the assays in MRC-5 but not in CEFs cells, we have hypothesised that HPV-L1 expression was mainly limited to human cells due to the humanised HPV-L1 gene sequence that was used for the FP_L1 _recombinant construction. However, detection of HPV-L1 also in non-human primate Vero cells by Western blotting instead suggests that although present, only the non-native HPV-L1 structure can be detected in these cells. The absence of any specific band in FP_L1_-infected CEFs both by immunoprecipitation and Western blotting can be ascribed to the prevalent expression of the early and late genes of the vector and to its cytopathogenicity, which might prevent expression of the heterologous HPV-L1 transgene.

On the other hand, the HPV-L1 expression in CEFs determined by immunofluorescence performed 6 h p.i. demonstrates that HPV-L1 can also be detectable in replication-permissive cells, although only at early times of infection, when the vector is expressing non-structural enzymatic proteins with poor cytopathic effects. Indeed, HPV-L1 expression was sometimes also found in CEFs by immunoprecipitation, when the samples were loaded in excess (our unpublished data). This suggests early/low expression in these cells, which makes it rarely detectable.

Similarly, HPV-L1 capsid proteins never appear to be correctly structured as VLPs in CEFs, although they were present in Vero and MRC-5 cells. While it is not surprising that VLPs are not detectable in cells where the recombinant replicates, as vector protein synthesis might be detrimental to HPV-L1 production and VLP assembly, the reason for the low VLP production in Vero and MRC-5 cells is not completely clear. This cannot be ascribed to the L1 gene coding sequence inserted into FP_L1_, which, as with other constructs [31,49,50] that can produce VLPs, starts from the second ATG. Indeed, transcription from the first ATG prevents VLP formation (M. Mueller, personal communication).

As already described [51], L1 capsid proteins can self-assemble as single capsomers (pentamers), T1 particles (12 pentamers) or complete VLPs (T7, 72 pentamers). At the ultrastructural level, we demonstrated the presence of both T1 and T7 particles. Although these are present in limited amounts, this confirms that FP-driven L1 proteins can, at least in part, self-assemble correctly. Since the L1 protein produced by the FP_L1 _recombinant has the NLS, nuclear localization is expected, in spite of the vector transgene expression in the cytoplasm. T1 capsomers were found in the nucleus, which were still not assembled as complete VLPs. VLPs were seen in pseudo-vacuoles, although very rarely, as if they were in the cytoplasm as a result of the tangential section of a plasma membrane invagination. We suggest therefore that the VLPs in the pseudo-vacuoles might represent extracellular VLPs that were previously assembled in the nucleus and were then released by already lysed cells.

We hypothesise that the GFP gene co-inserted with the HPV-L1 transgene into the vector to track the expression of the recombinant, the expression of which is driven by the strong SP synthetic promoter, might interfere with HPV-L1 expression. Although other studies have also used constructs containing GFP for recombinant selection [31,52], the hypothesis that GFP co-espression might hamper high production of the heterologous gene and VLP assembly has already been investigated by K. Kindsmueller (Institute of Medical Microbiology and Hygene, University of Regensburg, Germany, personal communication). By removing the GFP gene, we also obtained higher gene expression using other recombinants produced in our laboratory (unpublished data).

The similar decrease over time in both HPV-L1 and GFP mRNA transcripts shown by real-time PCR does not correlate with the low amounts of VLP production. However, data from our laboratory with recombinants that co-express transgenes and GFP suggest that SP promoter activity and/or GFP expression might interfere negatively, by limiting the synthesis of the L1 protein and VLP assembly. This might justify the good mRNA expression for both of these transgenes up to 10 days p.i. with very low VLP amounts.

It is therefore likely that HPV-L1 protein expression, rather than mRNA, is responsible for the low VLP assembly, which can justify the good mRNA expression for both of these transgenes up to 10 days p.i. with very low VLP amounts.

Therefore, before animal immunization, it will be interesting to compare a new FP_L1 _construct lacking the SP-GFP complex with the present FP_L1 _double recombinant, both for the differences in mRNA and protein expression and for VLP production.

## Conclusions

As L1 appears to be correctly expressed both by replication restrictive and permissive cell lines, the FP_L1 _recombinant might represent a new prophylactic vaccine, which can putatively cross-present the antigen in association with both MHC class I and II complexes. This might contribute to a complete humoral and cellular immunity when compared to the commercially available vaccines. L1 expression was observed for almost three weeks, and this is a pre-requisite for a durable immune response. The limited amounts of assembled VLPs seen by electron microscopy make these data preliminary to the use of a modified construct for the analysis of the immune responses and protection in animal models. Although the currently available vaccines are efficient in eliciting a neutralizing immunity, the 7-year efficacy appears limited since oncogenicity is often revealed only after decades of incubation. An alive non-replicating vaccine might fit the purpose better if it can produce the L1 protein and VLPs.

## Competing interests

The authors declare that they have no competing interests.

## Authors' contributions

CZ performed the molecular cloning and RT-PCR, prepared the primary cell cultures, revised the Figures, and analysed the data and the study results; EP performed animal immunisations, Western blotting, real-time PCR, and prepared Figures; SP performed the production of the poxvirus and the HPV-L1 recombinant; MB performed the immunofluorescence assays, and the poxvirus recombinant purification; CDGM performed electron microscopy, and conceptualised, designed, and supervised the whole study; AR performed the immunoprecipitation assays, assisted in the animal immunisations, analysed the data, interpreted the study results, and prepared the manuscript. All of the authors have read and approved the present version of the manuscript.
